# Predicting survival in patients with myelodysplastic/myeloproliferative neoplasms with *SF3B1* mutation and thrombocytosis

**DOI:** 10.1038/s41375-024-02262-2

**Published:** 2024-05-07

**Authors:** Fuhui Li, Tiejun Qin, Bing Li, Shiqiang Qu, Lijuan Pan, Peihong Zhang, Qi Sun, Wenyu Cai, Qingyan Gao, Meng Jiao, Junjie Li, Xiaofei Ai, Jiao Ma, Robert Peter Gale, Zefeng Xu, Zhijian Xiao

**Affiliations:** 1 State Key Laboratory of Experimental Hematology, National Clinical Research Center for Blood Diseases, Haihe Laboratory of Cell Ecosystem, Institute of Hematology and Blood Diseases Hospital, Chinese Academy of Medical Sciences & Peking Union Medical College, Tianjin, China; 2 MDS and MPN Centre, Institute of Hematology and Blood Diseases Hospital, Chinese Academy of Medical Sciences & Peking Union Medical College, Tianjin, China; 3 Hematologic Pathology Center, Institute of Hematology and Blood Diseases Hospital, Chinese Academy of Medical Sciences & Peking Union Medical College, Tianjin, China; 4 Centre for Haematology, Department of Immunology and Inflammation, Imperial College of Science, Technology and Medicine, London, UK

**Keywords:** Myelodysplastic syndrome, Myeloproliferative disease, Risk factors

## Abstract

We investigated data from 180 consecutive patients with myelodysplastic/myeloproliferative neoplasms with *SF3B1* mutation and thrombocytosis (MDS/MPN-*SF3B1*-T) who were diagnosed according to the 2022 World Health Organization (WHO) classification of myeloid neoplasms to identify covariates associated with survival. At a median follow-up of 48 months (95% confidence interval [CI] 35–61 months), the median survival was 69 months (95% CI 59–79 months). Patients with bone marrow ring sideroblasts (RS) < 15% had shorter median overall survival (OS) than did those with bone marrow RS ≥ 15% (41 months [95% CI 32–50 months] *versus* 76 months [95% CI 59–93 months]; *P* < 0.001). According to the univariable analyses of OS, age ≥ 65 years (*P* < 0.001), hemoglobin concentration (Hb) < 80 g/L (*P* = 0.090), platelet count (PLT) ≥ 800 × 10E + 9/L (*P* = 0.087), bone marrow RS < 15% (*P* < 0.001), the Revised International Prognostic Scoring System (IPSS-R) cytogenetic category intermediate/poor/very poor (*P* = 0.005), *SETBP1* mutation (*P* = 0.061) and *SRSF2* mutation (*P* < 0.001) were associated with poor survival. Based on variables selected from univariable analyses, two separate survival prediction models, a clinical survival model, and a clinical-molecular survival model, were developed using multivariable analyses with the minimum value of the Akaike information criterion (AIC) to specifically predict outcomes in patients with MDS/MPN-*SF3B1*-T according to the 2022 WHO classification.

## Introduction

Refractory anemia with ring sideroblasts associated with marked thrombocytosis (RARS-T) was proposed as a provisional entity in 2001 for the World Health Organization (WHO) classification of hematopoietic tumors and classified as “myelodysplastic/myeloproliferative neoplasm (MDS/MPN), unclassifiable” [1, 2]. RARS-T remained in the 2008 revision of the WHO classification [3–5]. In the 2016 revision, RARS-T was renamed MDS/MPN with ring sideroblasts and thrombocytosis (MDS/MPN-RS-T) and became a well-characterized, distinct MDS/MPN overlap entity [6, 7].

MDS/MPN-RS-T is characterized by ≥15% bone marrow ring sideroblasts (RS) and sustained thrombocytosis [6]. The median age at diagnosis is ~75 years [8–13]. Approximately 80% of patients have normal cytogenetics, and approximately one-half have *SF3B1* and *JAK2* mutations [14]. In those with *SF3B1* mutations, the percentage of patients with bone marrow RS specified for the diagnosis of MDS/MPN-RS-T was unchanged [6]. This is unlike myelodysplastic syndrome with ring sideroblasts (MDS-RS), where only 5% bone marrow RS is required in the presence of *SF3B1* mutation [6].

The median survival of people with MDS/MPN-RS-T is ~75 months [8, 10, 11]. Previous studies reported that age >80 years, abnormal cytogenetics, hemoglobin concentration (Hb) ≤ 100 g/L, and *ASXL1* and/or *SETBP1* mutations were correlated with worse survival [9–11]. There is no widely accepted survival prediction model.

Because of the high frequency of *SF3B1* mutations in MDS/MPN-RS-T and the correlation between *SF3B1* mutation and bone marrow RS, in the 2022 WHO classification, people with *SF3B1* mutation and <15% bone marrow RS were reclassified as myelodysplastic/myeloproliferative neoplasms with *SF3B1* mutation and thrombocytosis (MDS/MPN-*SF3B1*-T) [15–19].

Previous studies of MDS/MPN-RS-T were based on the 2008 or 2016 revision of the WHO classification and were restricted to people with ≥15% bone marrow RS [8–12, 20]. We aimed to describe the clinical and molecular landscapes of MDS/MPN-*SF3B1*-T patients, compare the prognoses of people with < or ≥15% bone marrow RS, and develop prognostic models.

## Materials and methods

### Subjects

MDS/MPN-*SF3B1*-T was diagnosed according to the 2022 WHO classification of myeloid neoplasms [19]. A total of 180 consecutive patients with MDS/MPN-*SF3B1*-T in our hospital between June 2006 and August 2023 were enrolled in the study. We interrogated clinical (sex, age at diagnosis, date of leukemia transformation, and date of death or last follow-up) and laboratory data (blood cell count, bone marrow aspirate smear, bone marrow biopsy, bone marrow RS, karyotypes, and sequencing analysis) from the subjects at the time of diagnosis. Bone marrow aspirate smears at diagnosis were available for all subjects, and cytogenetic data were available and evaluable for 169 subjects. To explore the differences between our cohort and previous cohorts of MDS/MPN-RS-T patients, we compared clinical and laboratory parameters and molecular signatures between the Mayo–Moffitt cohort (patients with MDS/MPN-RS-T, *n* = 158) and our cohort (patients with MDS/MPN-*SF3B1*-T, *n* = 180) [11]. In our study, we developed two survival prediction models, namely, the clinical survival model and the clinical-molecular model. The clinical model was constructed using data from all patients in our cohort, designated training cohort 1 (*n* = 180). Moreover, a clinical-molecular model was generated based on patients with next-generation sequencing (NGS) information in our cohort, which was defined as training cohort 2 (*n* = 122). There was no significant difference in clinical or laboratory parameters between training cohort 1 and training cohort 2 (Supplementary Table S1). Data from 43 patients with MDS/MPN-RS-T in the International Prognostic Scoring System (IPSS)-Molecular cohort were obtained from the cBioPortal platform [21, 22]. This cohort, referred to as the validation cohort (*n* = 43), was used as an external dataset for validation. The study was approved by the Ethics Committees of the Institute of Hematology, Chinese Academy of Medical Science, and Peking Union Medical College according to the guidelines of the Declaration of Helsinki.

### Bone marrow evaluation

Wright‒Giemsa staining was performed on the bone marrow and blood slides of patients at the time of diagnosis. Five hundred nucleated cells from each bone marrow slide and 200 nucleated cells from each blood slide were enumerated and classified for histological assessment. Prussian blue staining was performed on bone marrow slides to identify and enumerate the RS. Hematoxylin–eosin and Gomori methenamine silver staining were routinely performed on bone marrow biopsy sections. The degree of bone marrow fibrosis was classified using European consensus guidelines [23].

### Cytogenetics

Chromosome analyses were performed on metaphase cells from unstimulated bone marrow aspirates after 24 h of culture using R-banding techniques. Cytogenetic abnormalities were analyzed and reported using the International System for Human Cytogenetic Nomenclature (2013) and classified according to the Revised International Prognostic Scoring System (IPSS-R) [24].

### Targeted gene sequencing

DNA from the bone marrow or peripheral blood of patients at diagnosis was used for NGS as described [25]. The sequences of 122 patients with MDS/MPN-*SF3B1*-T were sequenced with a 14-gene panel using NGS at diagnosis (Supplementary Table S2). In addition, in patients without NGS data, the *SF3B1* mutation (at the 666th and 700th amino acid sites) status in 28 patients with MDS/MPN-*SF3B1*-T was detected using Sanger sequencing, and the *JAK2 V617F* status in 42 patients with MDS/MPN-*SF3B1*-T was tested using allele-specific PCR with a sensitivity of 1%.

### Relative mutation dominance

In patients harboring both *SF3B1* and myeloproliferative neoplasm (MPN)-driver mutations (*JAK2/CALR/MPL* mutations) in MDS/MPN-*SF3B1*-T, a cutoff of ≥5% difference between variant allele frequencies (VAFs) of the two mutations was used to evaluate the relative dominant mutation [26]. Consequently, these patients were classified into three groups: *SF3B1* mutation dominance, MPN driver mutation dominance, and no dominance.

### Treatment and follow-up

The treatment regimens of 160 patients were documented during follow-up. A total of 111 (69.4%) patients received agents to improve anemia, including recombinant human erythropoietin, lenalidomide, androgens, cyclosporin or luspatercept; 17 (10.6%) patients received only cytoreductive agents, such as hydroxyurea or interferon; 25 (15.6%) patients received the above combination therapies; and 7 (4.4%) patients underwent watch and wait protocols. With a median follow-up of 48 months (95% confidence interval [CI] 35–61 months), the last follow-up was on March 16th, 2024, and 16 subjects (8.9%) were lost to follow-up. Thrombosis information and leukemia transformation status information were available for 100 and 126 subjects, respectively, according to medical records or telephone follow-up.

### Statistical analysis

Continuous variables were presented as medians and interquartile ranges (IQRs), and nominal variables were presented as counts and relative frequencies. Differences in continuous variables between the two groups were analyzed using the Mann–Whitney *U*-test. Nominal variables from different groups were compared using the Pearson chi-square test or Fisher’s exact test. Spearman’s correlation analysis was used to evaluate correlations between mutations in any two genes in the targeted NGS panel. *P* values were adjusted by Benjamini–Hochberg method in multiple comparisons. A two-tailed *P* value < 0.05 was considered significant. Overall survival (OS) was calculated from the date of diagnosis to the date of death or last follow-up. The median follow-up was calculated using the reverse Kaplan‒Meier method. Univariable analyses of survival were performed using the log-rank test, and variables with *P* values < 0.1 were selected for Cox multivariable analyses. In multivariable analyses, backward stepwise elimination was used for variable selection. Independent variables incorporated in the final survival models were based on the multivariable Cox regression model with the minimum Akaike information criterion (AIC) value in the training cohort [27–29]. Variables in the survival model were assigned weights based on the regression coefficients derived from the Cox regression model. Kaplan–Meier curves and Harrell’s concordance index (C-index) were used to assess the discrimination of survival models. Statistical analyses were performed with SPSS software version 26.0 (IBM Corp., Armonk, NY, USA), GraphPad Prism version 9.0 (GraphPad Inc., San Diego, CA, USA), and R software version 4.2.3 (R Foundation for Statistical Computing, Vienna, Austria).

## Results

### Clinical features and molecular landscape of MDS/MPN-*SF3B1*-T according to the 2022 WHO classification

There were 180 patients with MDS/MPN-*SF3B1*-T in our cohort. The median age of patients with MDS/MPN-*SF3B1*-T at diagnosis was 65 years (IQR, 57–70 years). The median Hb, median platelet count (PLT), and median bone marrow RS at diagnosis were 74 g/L (IQR, 63–88 g/L), 552 × 10E + 9/L (IQR, 487–718 × 10E + 9/L), and 38% (IQR, 24–57%), respectively. The clinical features and laboratory details at diagnosis of all patients with MDS/MPN-*SF3B1*-T were comprehensively documented in Table 1.

**Table 1:** Clinical and laboratory features in 180 patients with 2022 WHO-defined MDS/MPN-*SF3B1*-T, classified by BM RS percentage.

Variable	All patients with MDS/MPN-*SF3B1*-T (*n* = 180)	Patients with BM RS < 15% (*n* = 24)	Patients with BM RS ≥ 15% (*n* = 156)	*P* value
Males; *n* (%)	105 (58.3)	20 (83.3)	85 (54.5)	0.008
Age in years; median (IQR)	65 (57–70)	67 (61–71)	65 (57–70)	0.361
WBC × 10E + 9/L; median (IQR)	5.9 (4.2–8.8)	6.6 (3.7–9.0)	6.0 (4.2–8.8)	0.754
ANC × 10E + 9/L; median (IQR)	3.9 (2.2–5.6)	4.9 (2.1–6.3)	3.8 (2.2–5.5)	0.305
Hemoglobin g/dL; median (IQR)	74 (63–88)	75 (64–88)	74 (62–88)	0.719
MCV femtoliter; median (IQR)	Evaluable = 169	Evaluable = 21	Evaluable = 148	
	103.2 (96.5–109.5)	102.8 (90.4–110.2)	103.2 (96.5–109.3)	0.603
Platelets × 10E + 9/L; median (IQR)	552 (487–718)	630 (497–755)	540 (487–710)	0.176
lactic dehydrogenase U/L; median (IQR)	Evaluable = 126	Evaluable = 19	Evaluable = 107	
	216.4 (165.4–261.2)	300.5 (218.4–343.5)	204.2 (161.0–252.2)	0.002
Erythropoietin mIU/ml; median (IQR)	Evaluable = 138	Evaluable = 22	Evaluable = 116	
	248.2 (72.1–758.0)	586.7 (118.2–760.5)	212.6 (70.6–747)	0.422
Blasts in PB %; median (IQR)	0 (0)	0 (0)	0 (0)	1.000
Blasts in BM %; median (IQR)	0.5 (0–1.0)	1.0 (0–2.0)	0.5 (0–1.0)	0.114
BM RS %; median (IQR)	38 (24–57)	4 (1–10)	44 (28–60)	<0.001
reticulin fibrosis grade	Evaluable = 161	Evaluable = 22	Evaluable = 139	
reticulin fibrosis grade ≥ 2; *n* (%)	16 (9.9)	4 (18.2)	12 (8.6)	0.314
Cytogenetics	Evaluable = 169	Evaluable = 23	Evaluable = 146	
Abnormal karyotype; *n* (%)	34 (20.1)	3 (13.0)	31 (21.2)	0.528
Complex karyotype; *n* (%)	5 (3.0)	1 (4.3)	4 (2.7)	0.523
IPSS-R cytogenetics; *n* (%)				0.466
Very good	5 (3.0)	1 (4.3)	4 (2.7)	
Good	132 (78.1)	18 (78.3)	114 (78.1)	
Intermediate	27 (16.0)	3 (13.0)	24 (16.4)	
Poor	3 (1.8)	0 (0)	3 (2.1)	
Very poor	2 (1.2)	1 (4.3)	1 (0.7)	
Abdominal ultrasound	Evaluable = 84	Evaluable = 8	Evaluable = 76	
Splenomegaly (ultrasound); *n* (%)	41 (48.8)	5 (62.5)	36 (47.4)	0.658
Next-generation sequencing analysis; *n* (%)	Evaluable = 122	Evaluable = 23	Evaluable = 99	
*ASXL1*-mutated	21 (17.2)	3 (13.0)	18 (18.2)	0.778
*CALR*-mutated	0 (0)	0 (0)	0 (0)	/
*DNMT3A*-mutated	26 (21.3)	6 (26.1)	20 (20.2)	0.735
*IDH1*-mutated	1 (0.8)	0 (0)	1 (1.0)	1.000
*IDH2*-mutated	0 (0)	0 (0)	0 (0)	/
*JAK2*-mutated	40 (32.8)	5 (21.7)	35 (35.4)	0.210
*MPL*-mutated	8 (6.6)	2 (8.7)	6 (6.1)	1.000
*RUNX1*-mutated	2 (1.6)	0 (0)	2 (2.0)	1.000
*SETBP1*-mutated	8 (6.6)	3 (13.0)	5 (5.1)	0.354
*SF3B1*-mutated	114 (93.4)	23 (100)	91 (91.9)	0.346
*SRSF2*-mutated	6 (4.9)	3 (13.0)	3 (3.0)	0.143
*TET2*-mutated	36 (29.5)	4 (17.4)	32 (32.3)	0.207
*TP53*-mutated	10 (8.2)	4 (17.4)	6 (6.1)	0.173
*U2AF1*-mutated	2 (1.6)	0 (0)	2 (2.0)	1.000
Thrombotic event	Evaluable = 100	Evaluable = 11	Evaluable = 89	
Thrombotic event, at or prior to diagnosis; *n* (%)	15 (15)	3 (27.3)	12 (13.5)	0.447
Thrombotic event, after diagnosis; *n* (%)	4 (4)	0 (0)	4 (4.5)	1.000
Leukemic transformations	Evaluable = 126	Evaluable = 13	Evaluable = 113	
Leukemic transformations; *n* (%)	11 (8.7)	1 (7.7)	10 (8.8)	1.000

*WHO* World Health Organization, *MDS/MPN-SF3B1-T* myelodysplastic/myeloproliferative neoplasm with *SF3B1* mutation and thrombocytosis, *IQR* interquartile range, *BM* bone marrow, *RS* ring sideroblasts, WBC white blood cell count, *ANC* absolute neutrophil count, *MCV* mean corpuscular volume, *PB* peripheral blood, *IPSS-R* revised international prognostic scoring system.

**Fig. 1:**
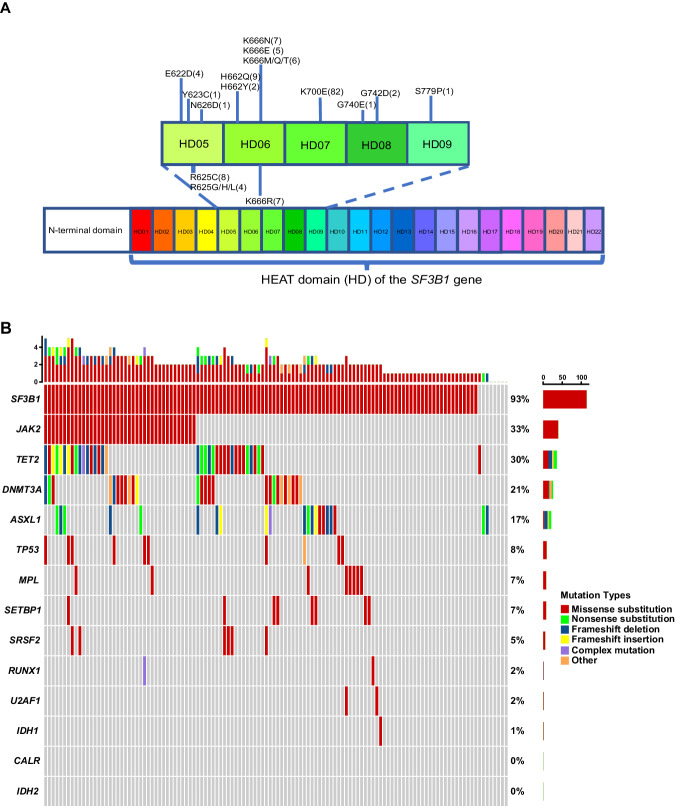
Molecular genetics signatures of patients with MDS/MPN-SF3B1-T. **A** Distribution of specific mutation sites in the *SF3B1* gene in 134 patients with *SF3B1* mutations detected by NGS or Sanger sequencing. There were 4 patients with 2 mutation sites and 1 patient with 3 mutation sites in *SF3B1;* therefore, a total of 140 mutations in *SF3B1* were detected in 134 *SF3B1*-mutated patients. B Molecular landscape of 122 patients with MDS/MPN-*SF3B1*-T detected by NGS. MDS/MPN-*SF3B1*-T myelodysplastic/myeloproliferative neoplasm with *SF3B1* mutation and thrombocytosis, NGS next-generation sequencing.

In our cohort, cytogenetic data were available and evaluable for 169 patients. A total of 135 (79.9%) patients harbored normal karyotypes and complex karyotypes were detected in 5 (3.0%) patients. The most common mutated gene in MDS/MPN-*SF3B1*-T was *SF3B1*. *SF3B1* mutations were detected in 134 (89.3%) patients, using either NGS or direct Sanger sequencing. The specific mutations of *SF3B1* included *K700E* (61.2%), *K666N/R/E/M/Q/T* (18.7%), *H625C/G/H/L* (9.0%) and *H662Q/Y* (8.2%) (Fig. 1A). Among the 122 patients who underwent NGS, the commonly mutated genes in MDS/MPN-*SF3B1*-T included *SF3B1* (93.4%), *JAK2* (32.8%), *TET2* (29.5%), *DNMT3A* (21.3%), *ASXL1* (17.2%), *TP53* (8.2%), *MPL* (6.6%), *SETBP1* (6.6%), and *SRSF2* (4.9%) (Fig. 1B and Supplementary Table S3). Correlation analysis of different gene mutations in MDS/MPN-*SF3B1*-T revealed that the correlation coefficients of mutations in any two genes were lower than 0.3 (all adjusted *P* > 0.05), indicating weak correlations (Supplementary Fig. S1). NGS revealed that 46 (37.7%) patients harbored both *SF3B1* and MPN driver mutations, 2 of whom had simultaneous mutations in *SF3B1*, *JAK2,* and *MPL*. Among patients with both *SF3B1* and MPN driver mutations, 22 (47.8%), 13 (28.3%) and 11 (23.9%) patients exhibited *SF3B1* mutation dominance, no dominance, and MPN driver mutation dominance, respectively (Supplementary Fig. S2).

Compared with patients diagnosed with MDS/MPN-RS-T within the Mayo–Moffitt cohort (*n* = 158), those diagnosed with 2022 WHO-defined MDS/MPN-*SF3B1*-T in our cohort (training cohort 1, *n* = 180) had a younger median age (65 *versus* 71 years), a lower median hemoglobin level (74 *versus* 95 g/L) and a greater percentage of *TET2* mutations (29.5% *versus* 7.0%, *P* = 0.003) at diagnosis (Supplementary Table S4) [11].

### Comparison of MDS/MPN-*SF3B1*-T patients with <15% bone marrow RS and ≥15% bone marrow RS

**Fig. 2:**
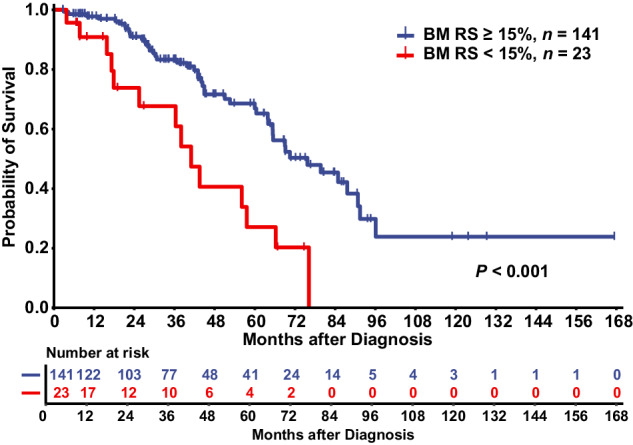
Comparison of survival between patients with ≥15% BM RS and those with <15% BM RS in MDS/MPN-*SF3B1*-T patients. BM bone marrow, RS ring sideroblasts, MDS/MPN-*SF3B1*-T myelodysplastic/myeloproliferative neoplasm with *SF3B1* mutation and thrombocytosis.

Compared with MDS/MPN-RS-T, one of the key modifications of the 2022 WHO classification for MDS/MPN-*SF3B1*-T was that patient with <15% bone marrow RS and *SF3B1* mutations were newly involved in the disease. We divided 180 patients with MDS/MPN-*SF3B1*-T into two groups: patients with <15% bone marrow RS (*n* = 24) and those with ≥15% bone marrow RS (*n* = 156). At least one *SF3B1* mutation was detected in 24 patients with <15% bone marrow RS using either NGS or Sanger sequencing. Patients with <15% bone marrow RS had a greater proportion of the male (83.3% *versus* 54.5%, *P* = 0.008) and had a higher median lactic dehydrogenase level (300.5 U/L [IQR, 218.4–343.5 U/L] *versus* 204.2 U/L [IQR, 161.0–252.2 U/L], *P* = 0.002). No significant differences in age at diagnosis, blood cell count, karyotype stratification, or molecular landscape were noted between the two groups (Table 1). One patient with <15% bone marrow RS and 15 patients with ≥15% bone marrow RS were lost to follow-up. The median OS of patients with <15% BM RS (*n* = 23; 41 months, 95% CI 32–50 months) was obviously inferior to that of patients with ≥15% BM RS (*n* = 141; 76 months, 95% CI 59–93 months; *P* < 0.001) (Fig. 2). Furthermore, we also divided patients with <15% bone marrow RS and *SF3B1* mutations into another two groups according to 5% bone marrow RS and found no difference in survival for patients with 5–15% BM RS (*n* = 12; median OS 41 months, 95% CI 31–50 months) compared with those with <5% BM RS (*n* = 11; median OS 38 months, 95% CI 6–70 months; *P* = 0.905; Supplementary Fig. S3).

### Prognostic factors for overall survival in MDS/MPN-*SF3B1*-T patients

The median OS of all patients with MDS/MPN-*SF3B1*-T was 69 months (95% CI 59–79 months). Leukemia transformations and thrombotic events after diagnosis were documented in 11 patients (8.7%) and 4 (4.0%) patients, respectively.

Univariable analyses of OS showed that age ≥ 65 years (*P* < 0.001), Hb < 80 g/L (*P* = 0.090), PLT ≥ 800 × 10E + 9/L (*P* = 0.087), bone marrow RS < 15% (*P* < 0.001), IPSS-R cytogenetic category intermediate/poor/very poor (*P* = 0.005), *SETBP1* mutation (*P* = 0.061) and *SRSF2* mutation (*P* < 0.001) were associated with poor survival (Supplementary Table S5).

**Fig. 3:**
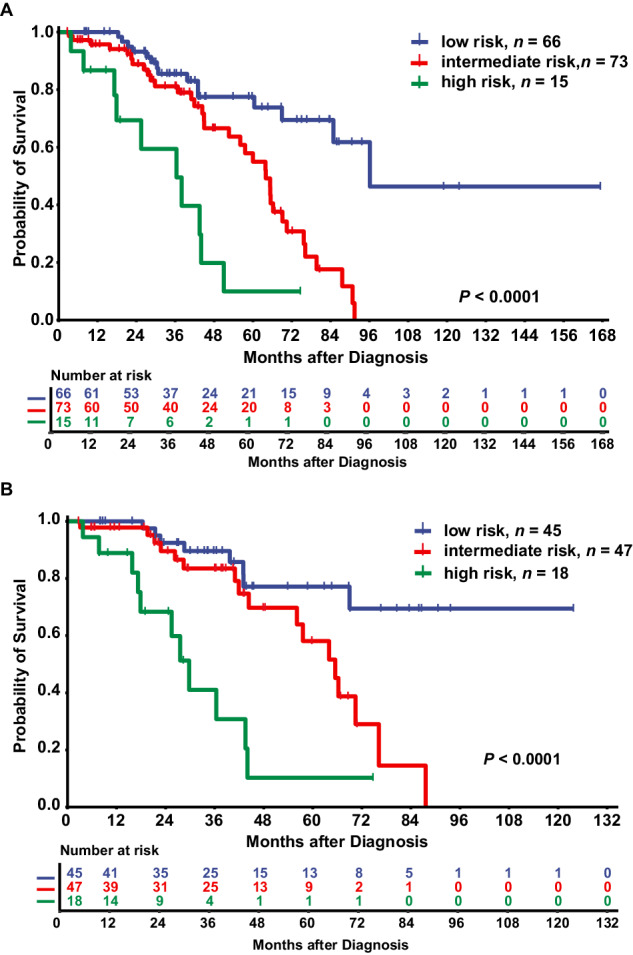
Kaplan–Meier probability estimates of OS in training cohort 1 and training cohort 2. **A** Kaplan–Meier probability estimates of OS in training cohort 1 (*n* **=** 180) were presented across the clinical survival model risk categories. The cytogenetic information or survival status of 26 patients in training cohort 1 was not available. Thus, 154 patients were included in the clinical survival model. Patients were divided into three categories: low (median OS 96 months, 95% CI unavailable), intermediate (median OS 64 months, 95% CI 56–72 months) and high risk (median OS 36 months, 95% CI 18–55 months) (low *versus* intermediate risk, *P* < 0.001; low *versus* high risk, *P* < 0.001; intermediate *versus* high risk, *P* = 0.001). **B** Kaplan–Meier probability estimates of OS in training cohort 2 (*n* = 122) were presented across the clinical-molecular survival model risk categories. The cytogenetic information and survival status of 12 patients were not available for training cohort 2. Thus, 110 patients were included in the clinical-molecular survival model. Patients were divided into three categories: low (median OS not reached), intermediate (median OS 65 months, 95% CI 55–76 months) and high risk (median OS 30 months, 95% CI 23–36 months) (low *versus* intermediate risk, *P* = 0.011; low *versus* high risk, *P* < 0.001; intermediate *versus* high risk, *P* < 0.001). OS overall survival.

In training cohort 1 (*n* = 180), age ≥ 65 years, Hb < 80 g/L, PLT ≥ 800 × 10E + 9/L, bone marrow RS < 15%, and IPSS-R cytogenetic category intermediate/poor/very poor were included in the multivariable analysis (Supplementary Table S5). The multivariable Cox regression model with the minimum AIC value included all five variables mentioned above. Considering these five variables, we developed a clinical survival model in which age ≥ 65 years, Hb < 80 g/L, PLT ≥ 800 × 10E + 9/L, bone marrow RS < 15%, and IPSS-R cytogenetic category intermediate/poor/very poor were worth 1 point, 1 point, 1 point, 2 points, and 1 point, respectively (Table 2). Then, we classified patients into three survival categories: low (0–1 point, *n* = 66, median OS 96 months, 95% CI unavailable), intermediate (2–3 points, *n* = 73, median OS 64 months, 95% CI 56–72 months) and high risk (≥4 points, *n* = 15, median OS 36 months, 95% CI 18–55 months), among which survival was significantly different (low risk *versus* intermediate risk, *P* < 0.001; low risk *versus* high risk, *P* < 0.001; intermediate risk *versus* high risk, *P* = 0.001; Fig. 3A).

**Table 2:** Variables assignment in clinical survival model and clinical-molecular survival model.

Survival model	Variables	*β*	*HR* (95%CI)	Score assigned	Risk category
Clinical survival model	Age ≥ 65 years	0.877	2.405(1.335–4.334)	1	0-1: low risk 2–3: intermediate risk ≥4: high risk
	IPSS-R cytogenetic category intermediate/poor/very poor	0.715	2.044 (1.072–3.896)	1	
	Hemoglobin < 80 g/L	0.577	1.780 (1.013–3.126)	1	
	Platelet count ≥ 800 × 10E + 9/L	0.679	1.971(1.065–3.648)	1	
	BM RS < 15%	1.218	3.381 (1.777–6.433)	2	
Clinical-molecular survival model	Age ≥ 65 years	0.599	1.820 (0.823–4.025)	1	0–1: low risk 2–3: intermediate risk ≥4: high risk
	IPSS-R cytogenetic category intermediate/poor/very poor	0.814	2.256 (0.919–5.542)	1	
	Hemoglobin < 80 g/L	0.561	1.752 (0.806–3.811)	1	
	Platelet count ≥ 800 × 10E + 9/L	0.682	1.978 (0.883–4.429)	1	
	BM RS < 15%	1.230	3.419 (1.600–7.305)	2	
	*SETBP1* mutation	1.353	3.869 (1.056–14.172)	2	
	*SRSF2* mutation	1.562	4.770 (1.311–17.350)	3	

*β* regression coefficients, *HR* hazard ratio, *CI* Confidence Interval, *IPSS-R* revised international prognostic scoring system, *BM* bone marrow, *RS* ring sideroblasts.

In training cohort 2 (*n* = 122), age ≥ 65 years, Hb < 80 g/L, PLT ≥ 800 × 10E + 9/L, BM RS < 15%, IPSS-R cytogenetic category intermediate/poor/very poor, *SRSF2* mutation and *SETBP1* mutation were included in the subsequent multivariable analyses based on the results of the preceding univariable analyses (Supplementary Table S5). The Cox proportional hazards regression model including all seven variables showed the lowest AIC value. We developed another clinical–molecular survival model including the above seven variables (Table 2) and classified patients in training cohort 2 into three survival categories: low risk (0–1 point, *n* = 45, median OS not reached), intermediate risk (2–3 points, *n* = 47, median OS 65 months, 95% CI 55–76 months) and high risk (≥4 points, *n* = 18, median OS 30 months, 95% CI 23–36 months), among which survival was significantly different (low risk *versus* intermediate risk, *P* = 0.011; low risk *versus* high risk, *P* < 0.001; intermediate risk *versus* high risk, *P* < 0.001) (Fig. 3B).

To compare the discriminative power of the clinical and clinical-molecular survival models, we also applied the clinical survival model to training cohort 2 and found that the clinical survival model could stratify patients in training cohort 2 into three risk categories with significantly different survival rates (Supplementary Fig. S4). In training cohort 2, the discriminative power of the clinical-molecular survival model, as measured using the C-index, was superior to that of the clinical survival model (C-index, 0.722 [±0.051] *versus* 0.695 [±0.053]).

We applied the clinical survival model and clinical-molecular survival model to the validation cohort. OS data were available for 41 patients in the validation cohort. Although subjects in the validation cohort classified as high risk were rare, survival among different risk categories was significantly different according to both the clinical survival model (*P* = 0.027, Supplementary Fig. S5A) and the clinical–molecular survival model (*P* = 0.013, Supplementary Fig. S5B). The clinical-molecular survival model also performed better than the clinical survival model in the validation cohort (C-index, 0.688 [±0.059] *versus* 0.677 [±0.059]).

## Discussion

MDS/MPN-RS-T is characterized by bone marrow RS ≥ 15% and sustained thrombocytosis with the proliferation of atypical megakaryocytes; these features partly overlap with those of MDS-RS and essential thrombocytosis [8, 14, 30, 31]. We compared clinical signatures and molecular landscapes between our cohort and the Mayo–Moffitt cohort and detected differences in several clinical parameters and *TET2* mutation frequencies (Supplementary Table S4) [11]. The mutational landscapes of 75 patients in another cohort of 92 RARS-T patients were described, and the frequency of *TET2* mutations was 25.3%, which was comparable to that in our cohort [20]. The difference in clinical and molecular genetic signatures between the Mayo–Moffitt cohort and our cohort may be partly due to differences in the ethnic groups. The *SF3B1* mutation is the most common mutation in the disease entity, and nearly 90% of patients with sequencing information in our study harbored the mutation. The specific mutation sites of *SF3B1* in our study were mainly distributed at the 700th, 666th, 625th, and 662nd amino acid sites, which was similar to the findings of other studies on RARS-T or MDS/MPN-RS-T [9–11]. Previous studies on RARS-T or MDS/MPN-RS-T reported that ~30–75% of patients have both *SF3B1* and *JAK2* mutations [9–11, 20, 32]. Co-mutations of *SF3B1* and *JAK2* or/and *MPL* mutations were present in 37.7% of patients who underwent NGS in our cohort, which is a relatively low percentage compared with that in some previous studies. This finding may be partly due to a lack of NGS information for some patients. *CALR* mutations are infrequent in MDS/MPN-RS-T patients, and *CALR* mutations were not detected in any of the patients in our cohort [10, 20, 33]. Several studies on MDS/MPN-RS-T have shown that the *SF3B1* mutation is more ancestral than MPN driver mutations according to comparisons of the VAFs of these mutations or Sanger sequencing of individual colonies [26, 31, 34]. In our study of patients with co-mutation of *SF3B1* and MPN driver mutations, we found that the VAF of *SF3B1* mutations was greater than (a cutoff of at least 5%) or equal to the VAF of *JAK2* or *MPL* mutations in almost 80% of patients, indicating that *SF3B1* mutations might occur before *JAK2* or *MPL* mutations in most patients with MDS/MPN-*SF3B1*-T [26].

The 2022 WHO classification of myeloid neoplasms revised the diagnostic criteria for MDS/MPN-RS-T based on *SF3B1* mutation and was renamed MDS/MPN-*SF3B1*-T [19]. The greatest change in the criteria was that patients with <15% bone marrow RS and *SF3B1* mutations were included in the disease entity. To the best of our knowledge, few studies have investigated patients with <15% bone marrow RS in MDS/MPN-*SF3B1*-T. Comparisons between patients with <15% bone marrow RS and those with ≥15% bone marrow RS illustrated that survival in the former subgroup was significantly inferior to that in the latter subgroup. Although the number of patients with <15% bone marrow RS was relatively low, heterogeneity between the two groups was obvious.

In studies related to MDS/MPN, unclassifiable (MDS/MPN-U), MDS/MPN-U patients with ≥15% bone marrow RS who did not meet the MDS/MPN-RS-T criteria were defined as MDS/MPN-U patients with bone marrow RS ≥ 15% (MDS/MPN-U-RS) [11, 35]. The aforementioned studies indicated that MDS/MPN-U-RS had similar outcomes to MDS/MPN-RS-T, whereas the survival of MDS/MPN-U patients with <15% bone marrow RS was inferior to that of MDS/MPN-RS-T patients, suggesting that bone marrow RS ≥ 15% might predict favorable outcomes in MDS/MPN-U patients [11, 35]. In our cohort of MDS/MPN-*SF3B1*-T patients, bone marrow RS ≥ 15% was also considered a favorable prognostic factor for survival, whereas *SF3B1* mutation was not confirmed to have prognostic significance. Previous studies have demonstrated that the molecular landscape is very different among *SF3B1*-mutated MDS, MDS/MPN and acute myeloid leukemia patients and that co-mutations contribute to diverse clinical and morphological features [31, 36]. Although it has been proven that *SF3B1* mutation can define a specific category in MDS, *SF3B1* mutation-based criteria in MDS/MPN-*SF3B1*-T still need further study [19, 37].

Previously, there were some studies on patients with RARS-T or MDS/MPN-RS-T that aimed to explore prognostic factors of survival [9–11]. Age > 80 years, wild-type *JAK2* and wild-type *SF3B1* were identified as the main adverse factors for survival in a cohort of 111 patients with RARS-T, whereas the prognostic significance of *SF3B1* and *JAK2* mutations has not been validated in other studies, including ours [9]. Another study on patients with RARS-T developed a hazard ratio weighted prognostic model based on abnormal karyotype, *ASXL1* and/or *SETBP1* mutations, and Hb < 100 g/L [10]. The above two studies enrolled patients based on the 2008 revision of the WHO classification of myeloid neoplasms [4]. A more recent study on 158 patients with MDS/MPN-RS-T was based on the 2016 WHO criteria, in which abnormal karyotype (excluding -Y) and Hb ≤ 100 g/L were found to be independent predictors of survival [11]. We developed a clinical survival model and clinical-molecular survival model using independent factors for the prediction of survival, which were determined not by a significant *P* value (*P* < 0.05) but by the minimum value of the AIC in multivariable Cox regression models [27, 38–40]. Compared with previous studies, such as the Mayo–Moffitt cohort published in 2022, we included patients with <15% bone marrow RS in the prognostic analysis of MDS/MPN-*SF3B1*-T for the first time [9–11]. We found that bone marrow RS < 15% was a predictor of adverse survival in patients with this disease. Thus, whether patients with <15% bone marrow RS and *SF3B1* mutations should be included in MDS/MPN-*SF3B1*-T needs further validation. *SRSF2* mutation (~50%) is one of the most frequent mutations in chronic myelomonocytic leukemia, but it has not been reported to be associated with prognosis [41]. However, previous studies have shown that *SRSF2* mutation predicts an adverse prognosis in patients with MDS [42]. A study on *SRSF2*-mutated neoplasms revealed that co-mutation of *SRSF2* and other splicing factors had a clear predominance of blastic phenotype [43]. In our cohort, *SF3B1* mutations were detected in all *SRSF2*-mutated patients (*n* = 6) (4 with the *SF3B1 K666E/T/M/R* mutation and 2 with the *SF3B1 K700E* mutation). Among the six patients with *SRSF2* mutations, one patient progressed to leukemia and subsequently died, whereas another three patients died from unknown causes. Based on the findings of previous studies and our data, we believe that *SRSF2* mutation may be associated with leukemia transformation and inferior survival in MDS/MPN-*SF3B1*-T patients. In addition, age, Hb, karyotype, and *SETBP1* mutation have been proposed as predictors of survival in other studies, whereas differences in the cutoff values of some variables may be attributed to differences in ethnic group or time of diagnosis [9–11]. A prior study reported that the frequency of thrombotic events in MDS/MPN-RS-T patients was comparable to that in patients with essential thrombocytosis but more frequent than that in patients with MDS-RS [8, 14]. In a Mayo Clinic cohort comprising 82 RARS-T patients, multivariable analysis of thrombosis-free survival revealed that the presence of *SF3B1* mutations independently predicted inferior thrombosis-free survival; however, the underlying mechanism involved remains unclear [44]. In our study, we did not identify significant factors for predicting thrombotic predisposition, potentially due to the limited documentation of thrombotic events and the short duration of follow-up.

The study is not devoid of limitations: (i) it involves a relatively small sample size and a brief follow-up period, (ii) it operates on a retrospective basis, and (iii) the external validation cohort comprises a relatively small number of patients and lacks those with <15% BM RS.

In conclusion, patients with <15% bone marrow RS and *SF3B1* mutations exhibited worse survival rates than those with ≥15% bone marrow RS in MDS/MPN-*SF3B1*-T patients. The inclusion of this patient group requires further validation. Additionally, we developed survival models for MDS/MPN-*SF3B1*-T patients based on the 2022 WHO classification, which should help physicians estimate survival in persons with MDS/MPN-*SF3B1*-T in the 2022 WHO classification.

### Supplementary information

Supplementary Material

## Data Availability

The datasets supporting the findings of this study are available from the corresponding authors upon reasonable request.
