# Supplementary material for: Predicting survival in patients with myelodysplastic/myeloproliferative neoplasms with SF3B1 mutation and thrombocytosis
Source: Leukemia. 2024 May 7;38(6):1334–41. doi: 10.1038/s41375-024-02262-2 (PMC11147759; doi:10.1038/s41375-024-02262-2)
Supplement: Supplementary file 1 — Supplementary Material [file 41375_2024_2262_MOESM1_ESM.docx]

**Supplement Table S1. Comparison of clinical and laboratory parameters between training cohort 1 and training cohort 2.**

| **Variable** | **Training cohort 1***  **（*n* = 180）** | **Training cohort 2***  **（*n* = 122）** | ***P* value** |
| --- | --- | --- | --- |
| Males; *n* (%) | 105 (58.3) | 75 (61.5) | 0.585 |
| Age in years; median (IQR) | 65 (57-70) | 64 (57-69) | 0.643 |
| WBC×10E+9/L; median (IQR) | 5.9 (4.2-8.8) | 6.1 (4.2-8.9) | 0.826 |
| ANC×10E+9/L; median (IQR) | 3.9 (2.2-5.6) | 4.0 (2.4-5.9) | 0.518 |
| Hemoglobin g/dL; median (IQR) | 74 (63-88) | 76 (66-90) | 0.500 |
| MCV femtoliter; median (IQR) | Evaluable = 169 | Evaluable = 113 |  |
|  | 103.2 (96.5-109.5) | 103.3 (95.7-109.5) | 0.993 |
| Platelets×10E+9/L; median (IQR) | 552 (487-718) | 573 (490-738) | 0.485 |
| lactic dehydrogenase U/L; median (IQR) | Evaluable = 126 | Evaluable = 101 |  |
|  | 216.4 (165.4-261.2) | 216.3 (166.7-261.3) | 0.995 |
| Erythropoietin mIU/ml;  median (IQR) | Evaluable = 138 | Evaluable = 111 |  |
|  | 248.2 (72.1-758.0) | 301.7 (72.8-758) | 0.856 |
| Blasts in PB %; median (IQR) | 0 (0) | 0 (0) | 1.000 |
| Blasts in BM %; median (IQR) | 0.5 (0-1.0) | 0.5 (0-1.5) | 0.746 |
| BM RS %; median (IQR) | 38 (24-57) | 34 (20-56) | 0.229 |
| reticulin fibrosis grade | Evaluable = 161 | Evaluable = 118 |  |
| reticulin fibrosis grade ≥ 2; *n* (%) | 16 (9.9) | 13 (11.0) | 0.770 |
| Cytogenetics | Evaluable = 169 | Evaluable = 115 |  |
| Abnormal karyotype; *n* (%) | 34 (20.1) | 22 (19.1) | 0.837 |
| Complex karyotype; *n* (%) | 5 (3.0) | 4 (3.5) | 1.000 |
| IPSS-R cytogenetics; *n* (%) | |  | 0.996 |
| Very good | 5 (3.0) | 4 (3.5) |  |
| Good | 132 (78.1) | 90 (78.3) |  |
| Intermediate | 27 (16.0) | 17 (14.8) |  |
| Poor | 3 (1.8) | 2 (1.7) |  |
| Very poor | 2 (1.2) | 2 (1.7) |  |
| Thrombotic event | Evaluable = 100 | Evaluable = 86 |  |
| Thrombotic event, at or prior to diagnosis; *n* (%) | 15 (15.0) | 13 (15.1) | 0.982 |
| Thrombotic event, after diagnosis; *n* (%) | 4 (4.0) | 3 (3.5) | 1.000 |
| Leukemic transformations | Evaluable = 126 | Evaluable = 97 |  |
| Leukemic transformations; *n* (%) | 11 (8.7) | 7 (7.2) | 0.511 |

*Training cohort 1, all patients in our cohort (*n* = 180); Training cohort 2, patients with NGS information in our cohort (*n* = 122).

Abbreviations: IQR, interquartile range; WBC, white blood cell count; ANC, absolute neutrophil count; MCV, mean corpuscular volume; PB, peripheral blood; BM, bone marrow; RS, ring sideroblasts; IPSS-R, Revised International Prognostic Scoring System; NGS, next-generation sequencing.

**Supplement Table S2. List of 14 genes included in the targeted NGS panel.**

| *ASXL1* | *CALR* | *DNMT3A* | *IDH1* | *IDH2* | *JAK2* | *MPL* |
| --- | --- | --- | --- | --- | --- | --- |
| *RUNX1* | *SETBP1* | *SF3B1* | *SRSF2* | *TET2* | *TP53* | *U2AF1* |

Abbreviations: NGS, next-generation sequencing.

**Supplement Table S3. The VAFs of different mutations in 122 patients with MDS/MPN-*SF3B1*-T detected by NGS.**

| **Molecular abnormalities** | ***n* (%)** | **VAF %; median (IQR)** |
| --- | --- | --- |
| *ASXL1* mutations | 21 (17.2) | 12.2 (5.5-33.2) |
| *CALR* mutations | 0 (0) | / |
| *DNMT3A* mutations | 26 (21.3) | 38.9 (31.9-46.3) |
| *IDH1* mutations | 1 (0.8) | 22.2 |
| *IDH2* mutations | 0 (0) | / |
| *JAK2* mutations | 40 (32.8) | 31.9 (17.3-43.0) |
| *MPL* mutations | 8 (6.6) | 27.4 (5.0-40.1) |
| *RUNX1* mutations | 2 (1.6) | 8.1 (5.7-10.5) * |
| *SETBP1* mutations | 8 (6.6) | 4.2 (1.6-39.5) |
| *SF3B1* mutations | 114 (93.4) | 39.3 (31.5-42.4) |
| *SRSF2* mutations | 6 (4.9) | 41.1 (19.8-44.8) |
| *TET2* mutations | 36 (29.5) | 33.1 (1.1-51.6) |
| *TP53* mutations | 10 (8.2) | 4.8 (4.1-44.2) |
| *U2AF1* mutations | 2 (1.6) | 27.0 (10.4-43.6) * |

Abbreviations: VAFs: variant allele frequencies; MDS/MPN-*SF3B1*-T, myelodysplastic/myeloproliferative neoplasm with *SF3B1* mutation and thrombocytosis; NGS, next-generation sequencing; IQR, interquartile range.

*Due to the lack of number of mutated-patients, median (range) was used for description.

**Supplement Table S4. Comparison of clinical features, laboratory features and molecular landscape between patients with MDS/MPN-*SF3B1*-T in our cohort (training cohort 1, *n* = 180) and patients with MDS/MPN-RS-T in the Mayo-Moffitt cohort (*n* = 158).**

| Variable | Training cohort 1  （*n* = 180） | Mayo-Moffitt cohort  （*n* = 158）* | *P* value |
| --- | --- | --- | --- |
| Males; *n* (%) | 105 (58.3) | 82 (52.0) | 0.235 |
| Age in years; median (range) | 65 (16-84) | 71 (38-94) | / |
| WBC x 10^9^/L; median (range) | 5.9 (1.5-45.6) | 7.6 (1.9-25.8) | / |
| ANC x 10^9^/L; median (range) | 3.9 (0.2-23.1) | / | / |
| Hemoglobin g/dL; median (range) | 74 (22-117) | 95 (66-145) | / |
| MCV femtoliter; median (range) | 103.2 (58.5-138.7) | / | / |
| Platelets x 10^9^/L; median (range) | 552 (450-1646) | 585 (454-1741) | / |
| lactic dehydrogenase U/L; median (range) | Evaluable = 126  216.4 (103.3-1312.7) | / | / |
| Erythropoietin mIU/ml; median (range) | Evaluable = 138  248.2 (8.1-797.0) | / | / |
| Blasts in PB %; median (range) | 0 (0) | / | / |
| Blasts in BM %; median (range) | 0.5 (0-4.5) | 1 (0-4) | / |
| BM RS %; median (range) | 38.0 (0-94.0) | 47.5 (15.0-90.0) | / |
| reticulin fibrosis grade | Evaluable = 161 |  | / |
| reticulin fibrosis grade ≥ 2; *n* (%) | 16 (9.9) | / | / |
| Cytogenetics | Evaluable = 169 | Evaluable = 72 |  |
| Abnormal karyotype; *n* (%) | 34 (20.1) | / | / |
| Abnormal karyotype (except -Y); *n* (%) | 32 (18.9) | 11 (15.0) | 0.497 |
| Complex karyotype; *n* (%) | 5 (3.0) | 2 (3.0) | 1.000 |
| IPSS-R cytogenetics; *n* (%)  Very good  Good  Intermediate  Poor  Very poor | 5 (3.0)  132 (78.1)  27 (16.0)  3 (1.8)  2 (1.2) | 5 (7.0)  59 (81.0)  4 (5.0)  3 (4.0)  2 (3.0) | 0.049 |
| Abdominal ultrasound | Evaluable = 84 | / | / |
| Splenomegaly (ultrasound); *n* (%) | 41 (48.8) |  |  |
| Next-generation sequencing analysis; *n* (%) | Evaluable = 122 |  |  |
| *ASXL1*-mutated | 21 (17.2) | Evaluable = 49; 10 (20) | 0.624 |
| *CALR*-mutated | 0 (0) | Evaluable = 43; 2 (5) | 0.067 |
| *DNMT3A*-mutated | 26 (21.3) | Evaluable = 43; 6 (14) | 0.294 |
| *IDH1*-mutated | 1 (0.8) | / | / |
| *IDH2*-mutated | 0 (0) | / | / |
| *JAK2*-mutated | 40 (32.8) | Evaluable = 43; 13 (30) | 0.758 |
| *MPL*-mutated | 8 (6.6) | / | / |
| *RUNX1*-mutated | 2 (1.6) | / | / |
| *SETBP1*-mutated | 8 (6.6) | Evaluable = 40; 4 (10) | 0.709 |
| *SF3B1*-mutated | 114 (93.4) | Evaluable = 111; 103 (93) | 0.845 |
| *SRSF2*-mutated | 6 (4.9) | Evaluable = 43; 1 (2) | 0.775 |
| *TET2*-mutated | 36 (29.5) | Evaluable = 43; 3 (7) | 0.003 |
| *TP53*-mutated | 10 (8.2) | Evaluable = 39; 1 (2) | 0.396 |
| *U2AF1*-mutated | 2 (1.6) | Evaluable = 43; 1 (2) | 1.000 |
| Leukemic transformations | Evaluable = 126 | Evaluable = 150 | 0.103 |
| Leukemic transformations; *n* (%) | 11 (8.7) | 6 (4) |  |

*Data of patients in Mayo-Moffitt cohort was cited from the work of Mangaonkar et al published in the “Blood Cancer Journal”. Reference: Mangaonkar AA, Lasho TL, Ketterling RP, Reichard KK, Gangat N, Al-Kali A, et al. Myelodysplastic/myeloproliferative neoplasms with ring sideroblasts and thrombocytosis (MDS/MPN-RS-T): Mayo-Moffitt collaborative study of 158 patients. Blood Cancer J 2022 Feb 1; 12(2): 26.

Abbreviations: MDS/MPN-*SF3B1*-T, myelodysplastic/myeloproliferative neoplasm with *SF3B1* mutation and thrombocytosis; MDS/MPN-RS-T, myelodysplastic/myeloproliferative neoplasm with ring sideroblasts and thrombocytosis; WBC, white blood cell count; ANC, absolute neutrophil count; MCV, mean corpuscular volume; PB, peripheral blood; BM, bone marrow; RS, ring sideroblasts; IPSS-R, Revised International Prognostic Scoring System.

**Supplement Table S5. Univariable and multivariable analyses of survival in MDS/MPN-*SF3B1*-T**

| Variable | Univariable analyses | Multivariable analyses (Training cohort 1*) | | Multivariable analyses  (Training cohort 2*) | | |
| --- | --- | --- | --- | --- | --- | --- |
|  | *P* value | *HR*（95% *CI*） | *P* value | | *HR*（95% *CI*） | *P* value |
| Age ≥ 65 years | < 0.001 | 2.405(1.335-4.334) | 0.003 | | 1.820 (0.823-4.025) | 0.139 |
| Complex karyotype | 0.370 |  |  | |  |  |
| IPSS-R cytogenetic category intermediate/poor/very poor | 0.005 | 2.044 (1.072-3.896) | 0.030 | | 2.256 (0.919-5.542) | 0.076 |
| Hemoglobin < 80g/L | 0.090 | 1.780 (1.013-3.126） | 0.045 | | 1.752 (0.806-3.811) | 0.157 |
| MCV ≥ 100fL | 0.818 |  |  | |  |  |
| Platelet count ≥ 800×10E+9/L | 0.087 | 1.971(1.065-3.648) | 0.031 | | 1.978 (0.883-4.429) | 0.097 |
| Absolute lymphocyte count < 1.2×10E+9/L | 0.999 |  |  | |  |  |
| serum ferritin ≥ 1000ng/ml | 0.839 |  |  | |  |  |
| Blasts in BM > 0.5% | 0.111 |  |  | |  |  |
| BM RS < 15% | < 0.001 | 3.381 (1.777-6.433) | < 0.001 | | 3.419 (1.600-7.305) | 0.002 |
| reticulin fibrosis grade ≥ 2 | 0.273 |  |  | |  |  |
| *SF3B1* mutation | 0.582 |  |  | |  |  |
| *JAK2* mutation | 0.708 |  |  | |  |  |
| *MPL* mutation | 0.700 |  |  | |  |  |
| *ASXL1* mutation | 0.600 |  |  | |  |  |
| *DNMT3A* mutation | 0.162 |  |  | |  |  |
| *IDH1* mutation | 0.499 |  |  | |  |  |
| *RUNX1* mutation | 0.618 |  |  | |  |  |
| *SETBP1* mutation | 0.061 |  |  | | 3.869 (1.056-14.172) | 0.041 |
| *SRSF2* mutation | < 0.001 |  |  | | 4.770 (1.311-17.350) | 0.018 |
| *TET2* mutation | 0.500 |  |  | |  |  |
| *TP53* mutation | 0.789 |  |  | |  |  |
| *U2AF1* mutation | 0.326 |  |  | |  |  |
| DTA mutation | 0.792 |  |  | |  |  |

*Training cohort 1, all patients diagnosed with MDS/MPN-*SF3B1*-T in our cohort (*n* = 180); training cohort 2, patients diagnosed with MDS/MPN-*SF3B1*-T with NGS information in our cohort (*n* = 122).

Abbreviations: MDS/MPN-*SF3B1*-T, myelodysplastic/myeloproliferative neoplasm with *SF3B1* mutation and thrombocytosis; IPSS-R, Revised International Prognostic Scoring System; MCV, mean corpuscular volume; BM, bone marrow; RS, ring sideroblasts; DTA, *DNMT3A*, *TET2* or *ASXL1*; HR, hazard ratio; CI, Confidence Interval; NGS, next-generation sequencing.


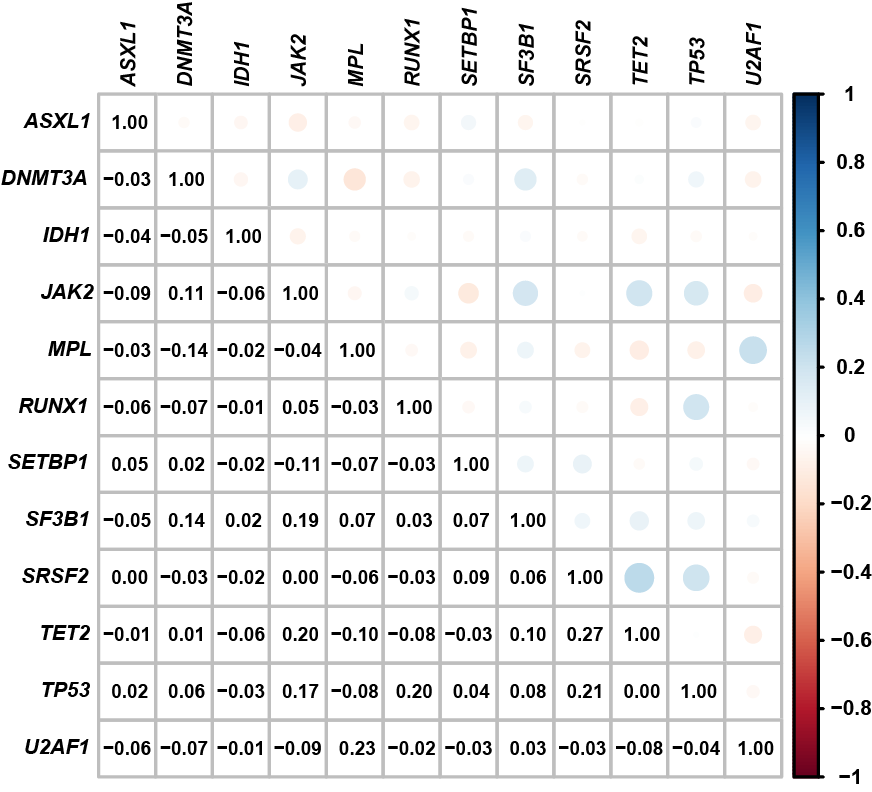


**Supplement Fig. S1** **Correlation analysis of different gene mutations in MDS/MPN-*SF3B1*-T.** Correlation analysis of different gene mutations in MDS/MPN-*SF3B1*-T revealed that the correlation coefficients of mutations in any two genes were lower than 0.3 (all adjusted *P* > 0.05).

Abbreviations: MDS/MPN-*SF3B1*-T, myelodysplastic/myeloproliferative neoplasm with *SF3B1* mutation and thrombocytosis.


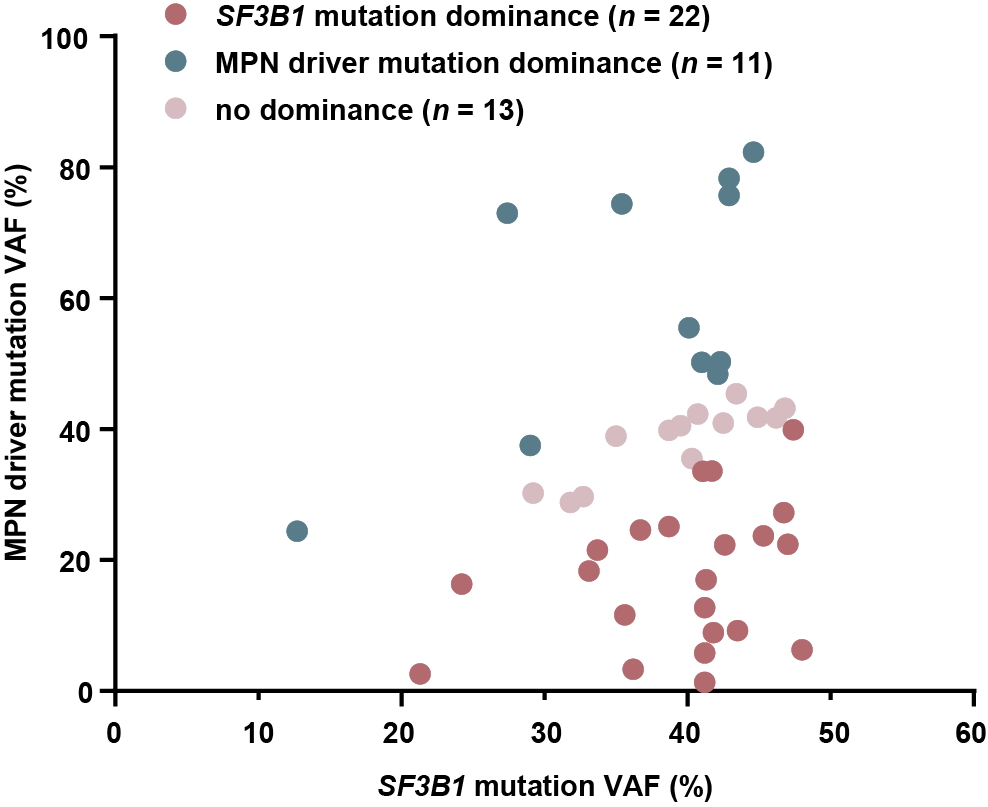


**Supplement Fig. S2 Comparison of VAF between *SF3B1* mutation and MPN driver mutation in 46 patients with cooccurrence of *SF3B1* mutation and MPN driver mutation in MDS/MPN-*SF3B1*-T.**

Abbreviations: VAF, variant allele frequency; MDS/MPN-*SF3B1*-T, myelodysplastic/myeloproliferative neoplasm with *SF3B1* mutation and thrombocytosis; MPN, myeloproliferative neoplasms.


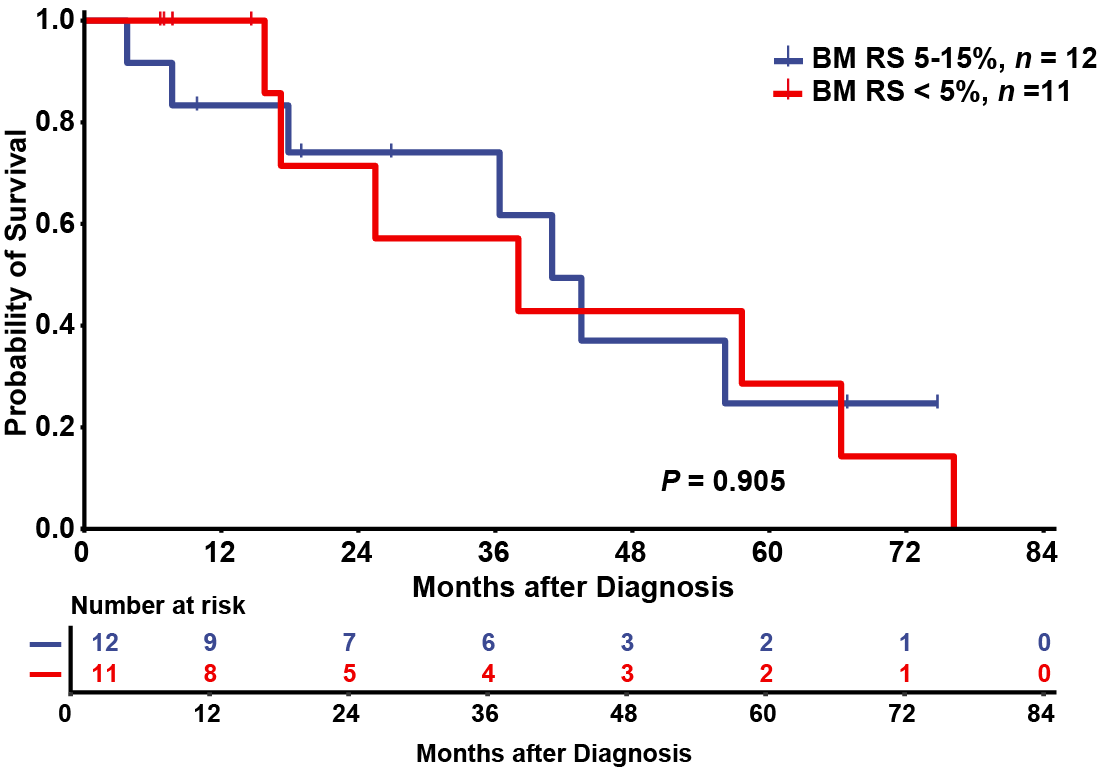


**Supplement Fig. S3** **Comparison of survival between patients with < 5% BM RS and those with 5-15% BM RS in MDS/MPN-*SF3B1*-T.**

Abbreviations: BM, bone marrow; RS, ring sideroblasts; MDS/MPN-*SF3B1*-T, myelodysplastic/myeloproliferative neoplasm with *SF3B1* mutation and thrombocytosis.


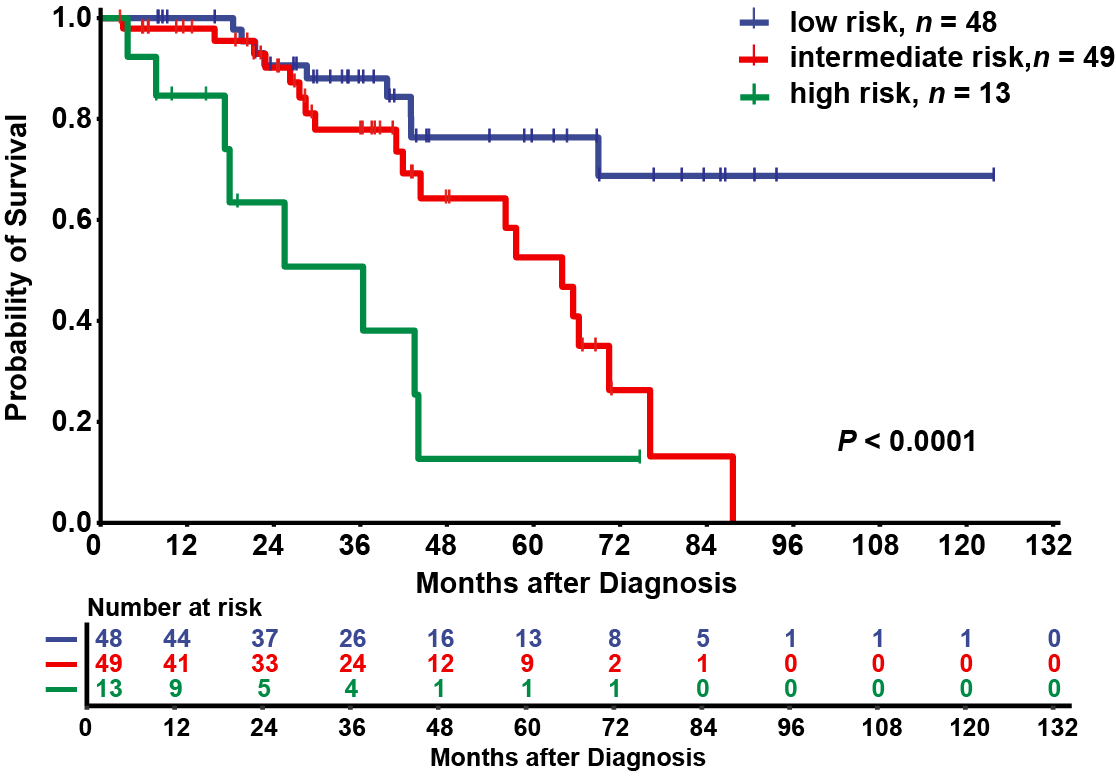


**Supplement Fig. S4** **Kaplan-Meier probability estimates of OS across clinical survival model risk categories in training cohort 2.** Cytogenetics information or survival status in 12 patients were not available in training cohort 2. Thus, there were 110 patients including in the clinical survival model risk categories. Patients were divided into three categories, low (median OS not reached), intermediate (median OS 64 months, 95% CI 53-75 months) and high risk (median OS 36 months, 95% CI 13-60months) (low versus intermediate risk, *P* = 0.005; low versus high risk, *P* < 0.001; intermediate versus high risk, *P* = 0.010).

Abbreviations: OS, overall survival; CI, Confidence Interval.


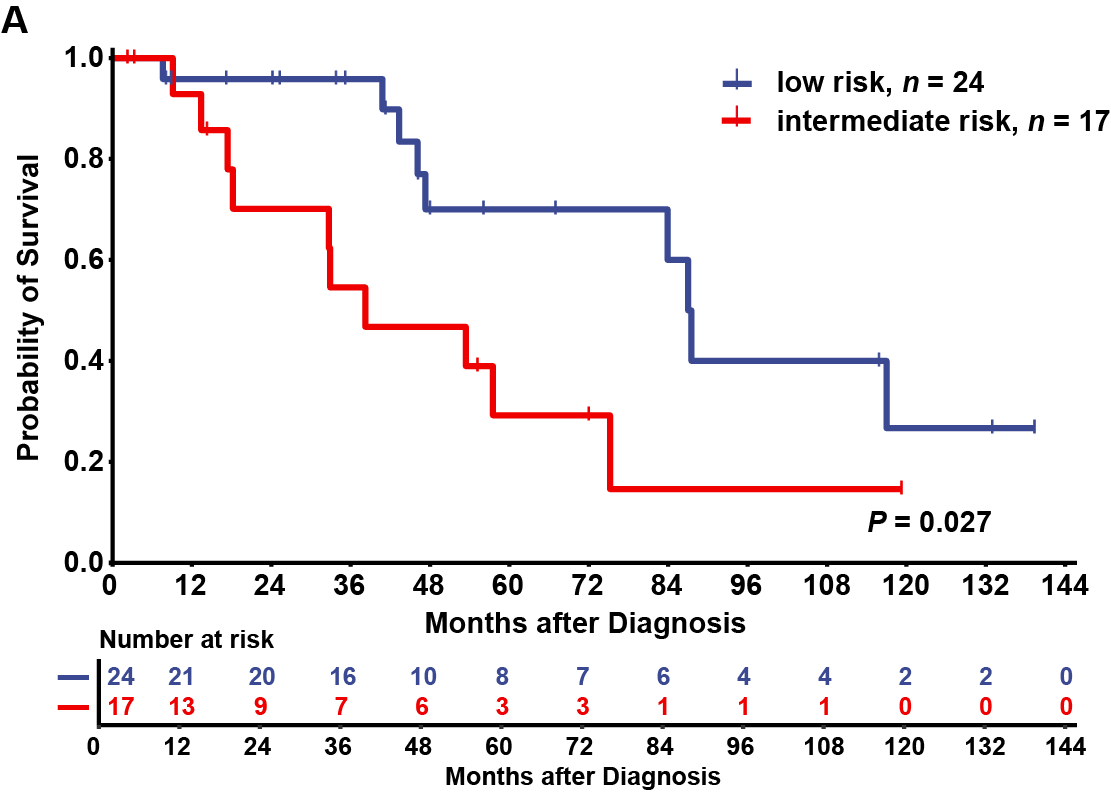


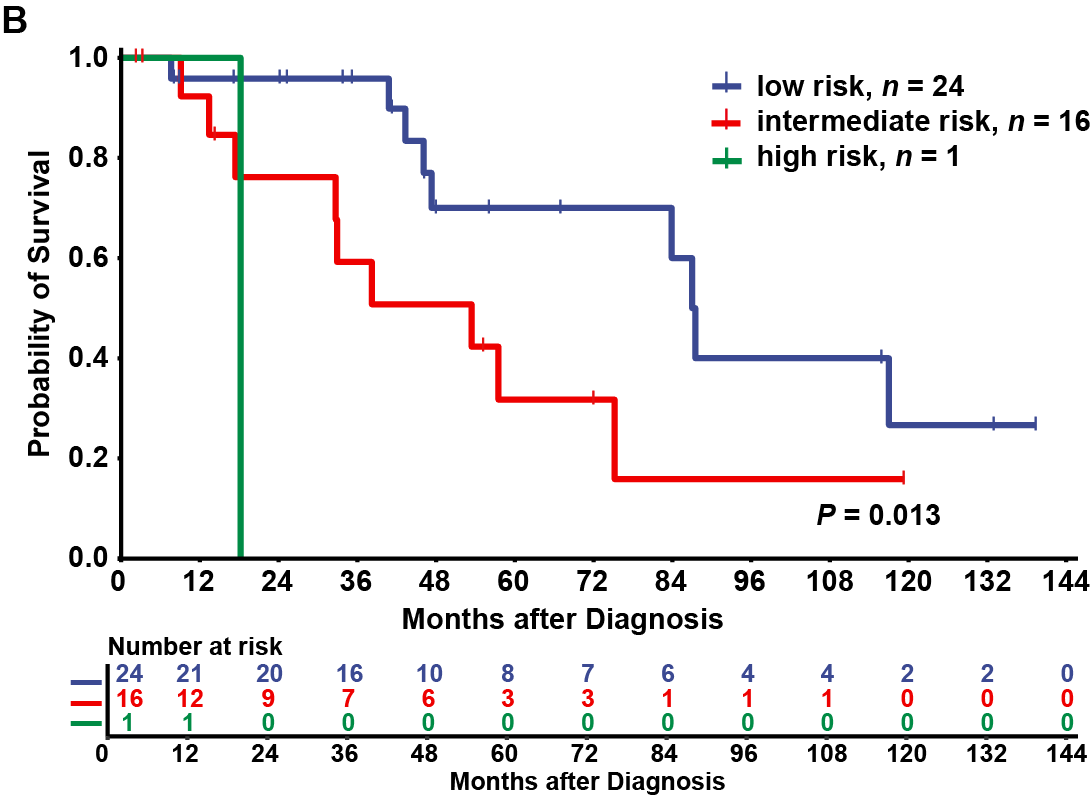


**Supplement Fig. S5** **Validation of clinical and clinical-molecular survival model on the validation cohort (*n* = 43)** (subjects with MDS/MPN-RS-T in IPSS-Molecular cohort* available through the cBioPortal platform). Survival data were available in 41 patients in the validation cohort.

1. **Kaplan-Meier probability estimates of OS across clinical survival model risk categories in the validation cohort.** Patients were divided into two categories (no patients were classified as high risk), low (median OS 88 months, 95% CI 82-93 months), intermediate (median OS 40 months, 95% CI 14-62 months) (low *versus* intermediate risk, *P* = 0.027).
2. **Kaplan-Meier probability estimates of OS across clinical-molecular survival model risk categories in the validation cohort.** Patients were divided into three categories, low (median OS 88 months, 95% CI 82-93 months), intermediate (median OS 53 months, 95% CI 19-88 months) and high risk (median OS 18 months) (low versus intermediate risk, *P* = 0.049; low versus high risk, *P* = 0.002; intermediate versus high risk, *P* = 0.235).

*Reference: Bernard E, Tuechler H, Greenberg PL, Hasserjian RP, Arango Ossa JE, Nannya Y, et al. Molecular International Prognostic Scoring System for Myelodysplastic Syndromes. NEJM Evid 2022 Jul; 1(7): EVIDoa2200008.

Abbreviations: MDS/MPN-RS-T, myelodysplastic/myeloproliferative neoplasm with ring sideroblasts and thrombocytosis; IPSS, International Prognostic Scoring system; OS, overall survival; CI, Confidence Interval.
